# Cerebral venous sinus thrombosis associated with cancer: analysis of the ACTION-CVT study

**DOI:** 10.1007/s11239-024-02997-w

**Published:** 2024-06-02

**Authors:** Maria Cristina Vedovati, Liqi Shu, Nils Henninger, Adeel S. Zubair, Mirjam R. Heldner, Sami Al Kasab, James E. Siegler, David S. Liebeskind, Kateryna Antonenko, Shadi Yaghi, Maurizio Paciaroni

**Affiliations:** 1 Department of Medicine and Surgery, University of Perugia, Perugia, Italy; 2 Department of Neurology, Brown University, Providence, RI USA; 3 Department of Neurology, University of Massachusetts Chan Medical School, Worcester, MA USA; 4 Department of Psychiatry, University of Massachusetts Chan Medical School, Worcester, MA USA; 5 Department of Neurology, Yale University, New Haven, CT USA; 6 Department of Neurology, Inselspital, University Hospital and University of Bern, Bern, Switzerland; 7 Department of Neurology, Medical University of South Carolina, Charleston, SC USA; 8 Department of Neurosurgery, Medical University of South Carolina, Charleston, SC USA; 9 Cooper Neurological Institute, Cooper University, Camden, NJ USA; 10 Department of Neurology, University of California at Los Angeles, Los Angeles, CA USA

**Keywords:** Direct oral anticoagulants, DOAC, Vitamin K antagonists, Cerebral venous thrombosis, Cancer

## Abstract

Nearly one fifth of patients with venous thromboembolism (VTE) have cancer. When both of these conditions occur, especially in cases of cerebral vein thrombosis (CVT), patient management is often challenging. The aim of this study was to compare the characteristics and event courses in patients affected by CVT with and without cancer. Consecutive patients with CVT from the ACTION-CVT cohort study were included if cancer status was reported. Risk factors as well as the clinical and radiological characteristics of patients were compared. Univariable and multivariable analyses were performed to assess variables associated with cancer. Kaplan-Meier method and log-rank test, logistic regression analysis, and propensity score matching were used to investigate any association between cancer-related CVT and study outcomes (primary outcome at 3-months: recurrent VTE or major hemorrhage; recurrent VTE; major hemorrhage; recanalization status; all-cause-death). Overall, 1,023 patients with CVT were included, of which 6.5% had cancer. Older age (adjusted odds ratio [aOR] 1.28 per decade increase; 95% confidence interval [CI] 1.08–1.52) and absence of headache (aOR 0.47; 95% CI 0.27–0.84) were independently associated with cancer. Patients with cancer had a higher risk of recurrent VTE or major hemorrhage (aOR 3.87; 95% CI 2.09–7.16), all-cause-death (aOR 7.56 95% CI 3.24–17.64), and major hemorrhage (aOR 3.70 95% CI 1.76–7.80). Recanalization rates, partial or complete, was not significantly different. CVT patients with cancer were more likely to be older, have no referred headache, and have worse outcomes compared to CVT patients without cancer.

**Supplementary Information:**

The online version contains supplementary material available at 10.1007/s11239-024-02997-w.

## Highlights

Among 1,023 patients with cerebral vein thrombosis from the ACTION-CVT study, 6.5% had cancer.

Older age and absence of headache were independently associated with cancer.

Cancer patients had worse outcomes.

**Graphical Abstract:**

**Supplementary Information:**

The online version contains supplementary material available at 10.1007/s11239-024-02997-w.

## Graphical abstract

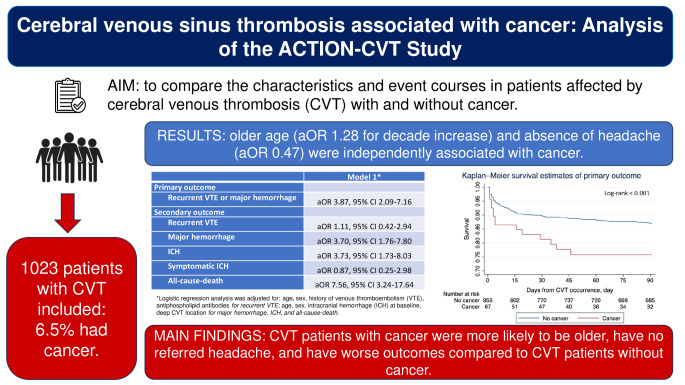

## Introduction

The risk of VTE in cancer patients has been reported to be four-fold higher that in the general population [1, 2] and about 20% of patients with VTE are found to have cancer at the time of VTE diagnosis [3]. Data from the International Study on Cerebral Vein and Dural Sinus Thrombosis (ISCVT), a prospective cohort study, reported that cancer accounts for 7.4% of cerebral vein thromboses (CVT) [4]. Specifically, cancer can promote CVT via local and systemic mechanisms which might include compression or invasion due to the tumor, induction of a hypercoagulable state, infection, paraneoplastics, as well as therapy related hematologic changes [5]. Moreover, CVTs can be the first symptom of a solid cancer, especially brain tumors and myeloproliferative neoplasms [6]. Whenever CVT occurs in elderly patients, the presence of malignancy or hematological disorders should be suspected [7].

The prognosis of CVT is generally favorable, despite the fact that a systematic review including 19 studies and 1,488 patients with CVTs reported that death within the first month occurred in 5.6% of patients and death at the end of follow-up was recorded to be 9.4% [8]. Most of the deaths were not considered to be a direct consequence of the CVT (24 out of 38 evaluated deaths during follow-up) [8].

Anticoagulant therapy is the mainstay treatment for CVTs [9, 10]. However, anticoagulation management of patients with cancer associated venous thrombosis is often complicated by an elevated risk of bleeding complications, especially in the presence of a brain tumor or thrombocytopenia [11, 12]. In light of this, the aim of this study was to compare the characteristics and event courses in patients affected by CVT with and without cancer.

## Methods

The methods and results of the ACTION-CVT study have been previously described in detail [13].

Institutional Review Board approval was obtained by each participating center before performing the study. De-identified data are available upon reasonable request to the corresponding Author.

### Patient population

The “*A*nti*c*oagula*tion* in the Treatment of *C*erebral *V*enous *T*hrombosis” (ACTION-CVT) study was an international, multicenter (USA, Italy, Switzerland, New Zealand) retrospective cohort study that included consecutive adult patients with CVT confirmed on brain imaging tests and admitted between January 1, 2015 up until December 31, 2020 [13]. Patients with CVT were initially identified using ICD-9 (325.0, 437.6, and 671.5) and ICD-10 codes (I67.6) with acceptable sensitivity and specificity [14, 15]. This was followed by a review of medical records and imaging studies to confirm the diagnosis of CVT. Only patients with available cancer status were included in this study.

### Study variables

The following variables were collected through medical record reviews, as described in the ACTION-CVT study [13]: demographic factors; CVT risk factors; clinical variables; imaging variables; laboratory variables closest to time of diagnosis; in-hospital treatments.

Cancer was defined as any malignant neoplasm other than non-melanoma skin cancers diagnosed at the time of CVT diagnosis or previously.

### Outcomes

The primary study outcome was defined as a composite of recurrent VTE and major hemorrhage. Other outcomes included recurrent VTE, major hemorrhage, intracranial hemorrhage (ICH), symptomatic ICH, all-cause-death, recanalization status, as well as the composite of recurrent VTE, major hemorrhage and all-cause-death.

Definitions of study outcomes have already been reported in the ACTION-CVT study [13], while here we report the main features:

- Recurrent VTE. Venous thromboembolic events occurring at any site were considered. Recurrent CVT was defined as de-novo CVT.
- Major hemorrhage was defined as new or worsening ICH or major extracranial hemorrhage. Major extracranial hemorrhage was defined as systemic hemorrhage with at least 2 g/dL drop in hemoglobin level or requiring blood transfusion occurring while on oral anticoagulation therapy.
- Symptomatic ICH was defined as any new or worsening intracranial hemorrhage on a follow-up brain imaging study causing new or worsening neurological signs or symptoms.
- Any death during follow-up.
- Imaging outcomes: any improvement in recanalization status after initiation of anticoagulation therapy on imaging study abstracted from radiology reports (no-recanalization, partial recanalization, or complete recanalization) [16].

Study outcomes were assessed at 3 and at 6 months.

### Analytical plan

Data verification was conducted by SY and LS with the aim of ensuring data integrity and consistency. All data used in the study was reviewed by the data coordinating center for missing fields, outliers, and incongruencies. Missing data were not imputed.

Categorical data were summarized in raw numbers and percentages, whereas continuous variables were presented using the number of patients, mean (standard deviation), or median (interquartile range). Baseline comparisons were performed by using Chi-squared test or Fisher’s exact test, T-test, or Wilcoxon rank sum test (Mann-Whitney two-sample) as appropriate. The reported p-values were derived from the results of two-sided tests. Risk factors, along with clinical and radiological characteristics of patients with and without cancer were compared. A multivariable analysis was performed to assess variables likely to be associated with cancer. The independent variables included in the multivariable analysis were selected from the univariable analysis using backward stepwise analysis with a *p* = 0.05 level as a screening criterion for selection of candidate variables.

For the outcome analysis the following were applied: unadjusted logistic-regression; logistic-regression adjusted for prespecified variables (Model 1); logistic-regression adjusted for both prespecified variables and variables that differed between the two groups (Model 2); propensity score matching. Using logistic regression, we calculated propensity scores based upon prespecified variables and headache as a clinical variable of interest. Moreover, radius matching with replacement and with a caliper distance of 0.05 were employed to match patients with active cancer to controls, conducted separately for each outcome due to varying prespecified variables.

Prespecified variables differed according to study outcome. For the recurrent VTE, we adjusted for variables associated with recurrent venous thrombosis as reported in prior studies: age, sex, history of VTE [17] as well as those that may biologically portend an increased risk, particularly the presence of one or more positive antiphospholipid antibodies. As for major hemorrhage, ICH, and all-cause-death, we adjusted for predictors of intracranial and/or extracranial hemorrhage: age, sex, ICH at baseline [18, 19], and deep CVT location; the latter being a known predictor of poor functional outcome and mortality in CVT [4].

Competing risk-adjusted models for predictors of primary outcome with mortality as a competing event were also assessed. The event course over time was assessed by Kaplan-Meier method and log-rank test. The primary outcome of interest was the composite of recurrent VTE or major hemorrhage at 3 months for both groups. Secondary outcomes of interest were recurrent VTE, major hemorrhage, recanalization status, ICH, and all-cause-death within 3 months.

Outcome analyses at 6 months, and major bleeding outcome excluding asymptomatic ICH at 3 and 6 months, were also performed.

Data are reported as odds ratios (OR) or as adjusted ORs at 95% confidential intervals (CI).

Data were analyzed using Stata (version 17.0) and a *p* < 0.05 was deemed to be statistically significant.

## Results

Among the 1,025 patients with CVT included in the original cohort, 2 lacked information on their cancer status and had to be excluded. Overall, 67 out of the 1,023 included patients (6.5%) had cancer. Table 1 summarizes the baseline characteristics of CVT patients with and without cancer.

On univariable analyses, patients with cancer were older, less frequently female, had lower body mass indexes (BMIs), along with higher rates of chronic kidney disease, liver disease, and were more likely to be active smokers (Table 1). With respect to CVT location, patients with cancer had significantly more frequent involvement of superficial veins.

Regarding the clinical presentation, headache was referred to be less frequent in patients with cancer. Duration of follow-up was significantly longer in patients without cancer (median 308 days) versus patients with cancer (median 112 days) (*p* < 0.001).

From multivariable analysis, older age was resulted being associated with the presence of cancer (adjusted OR 1.28 for each decade increase; 95% CI 1.08–1.52; *p* = 0.005) while patients with cancer were less likely to have headaches (adjusted OR 0.47; 95% CI 0.27–0.84; *p* = 0.010) (Table S1). After adjustment, the association of active smoking (adjusted OR 1.86; 95% CI 0.98–3.54; *p* = 0.059) and the involvement of superficial veins (adjusted OR 2.02; 95% CI 0.95–4.29; *p* = 0.067) with the presence of cancer were not statistically significant (Table S1).

## Associations between cancer and outcomes

**Fig. 1:**
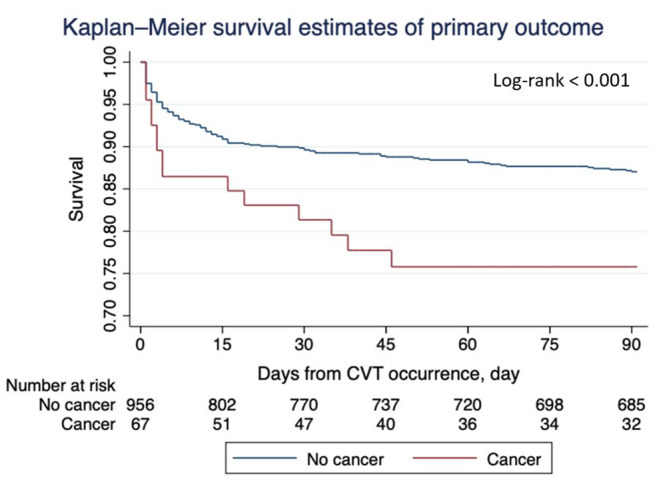
Survival from the composite of recurrent venous thrombosis or major hemorrhage in patients with and without cancer

The primary outcome occurred in 34.7 per 100 patient-years in patients with cancer versus 11.3 in those without (*p* < 0.001) (Fig. 1). From unadjusted logistic-regression analyses (Table 2), the presence of cancer was associated with a two-fold increased risk of primary outcome compared to those without cancer (OR 2.17; 95% CI 1.18–3.99; *p* = 0.012). After adjustment for the prespecified variables, the presence of cancer was associated with a more than 3-fold increased risk of primary outcome (adjusted OR 3.87, 95% CI 2.09–7.16 *p* < 0.001) (Model 1; Table 2). These results did not change after additionally adjusting for headache (adjusted OR 3.87; 95% CI 2.06–7.25 *p* < 0.001) (Model 2; Table 2). Likewise, similar results were recorded in the competing risk-adjusted models (Table S2).

**Fig. 2:**
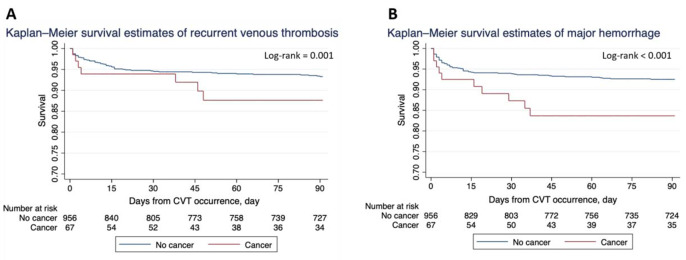
**A** Survival from recurrent venous thrombosis in patients with and without cancer; **B** Survival from major hemorrhage in patients with and without cancer

Recurrent venous thromboembolism occurred in 17.4 per 100 patient-years in patients with cancer versus 6.5 per for those without cancer (*p* = 0.001) (Fig. 2A). Similar results were observed between the two groups after adjusting for prespecified variables (Table 2).

Major hemorrhage occurred in 21.7 per 100 patient-years in patients with cancer versus 5.8 in those without cancer (*p* < 0.001) (Fig. 2B). Any ICH occurred in 19.5 per 100 patient-years in patients with cancer versus 5.2 per 100 patient-years in those without cancer (*p* < 0.001). Symptomatic ICH occurred in 4.3 per 100 patient-years in patients with cancer versus 2.3 in those without (*p* = 0.332). On unadjusted logistic regression and after adjustment for prespecified variables or variables that differed between the two groups, the presence of cancer was resulted being associated with a risk of major hemorrhage, when compared to patients without cancer (Table 2).

Death at 3 months occurred in 56.4 per 100 patient-years in patients with cancer versus 3.1 in those without cancer (*p* < 0.001). In unadjusted logistic regression analyses, the presence of cancer was associated with a higher risk of death (OR 14.90; 95% CI 7.55–29.43; *p* < 0.001). Finally, cancer remained associated with a significantly increased overall risk of death after adjusting for the different variables (Model 1 and Model 2 at Table 2).

Recanalization rates at 3 months were not significantly different for cancer patients compared to patients without cancer (58.8% [10 out of 17] vs. 73.0% [205 out of 281], OR 0.53, 95% CI 0.19–1.44; *p* = 0.213). No significant differences were observed after adjusting for the variables included in Models 1 and 2 (Table 2).

At 3-month follow-up, the following were recorded for the cancer group while on anticoagulant treatment: one recurrent VTE, one ICH, and nine deaths. Whereas, for the non-cancer group the following occurred: 35 recurrent venous thromboembolic events, 34 major hemorrhages (28 ICH) and 16 deaths.

The results of the analyses at 6 months are reported in Supplement (Table S3). Recanalization rates at 6 months were 66.7% (10/15) for the cancer group and 79.4% (227/286) for the non-cancer group (Table S3).

Regarding primary and secondary study outcomes at 3 and 6 months, similar results were observed when the use of anticoagulant treatment with vitamin K antagonist (VKA) was added to the Model.

### Propensity score matching

Propensity score matching with replacement included: 835 patients for primary outcome analysis, 1,018 patients for safety outcome and for death analyses, and 254 patients for recanalization outcome. Propensity score matching was effective as all the t-test p-values reached a *p* > 0.05, suggesting no significant post-matching differences.

Concerning, the results of the 3-month analyses, the presence of cancer was associated with higher risks of primary outcome (adjusted OR 2.93; 95% CI 1.32–6.52, *p* = 0.008), death (adjusted OR 11.33 95% CI 5.08–25.25, *p* < 0.001), major hemorrhage (adjusted OR 3.56; 95% CI 1.48–8.58, *p* < 0.001), and ICH (adjusted OR 3.56, 95% CI 1.42–8.91, *p* = 0.007) (Table 2). Table S4 summarizes the propensity score matching analysis findings at 6 months. Here, the primary study outcome, major hemorrhage, ICH, all-cause-death, the composite of recurrent VTE, major hemorrhage and death were significantly higher in cancer patients. Recanalization rates were not significantly different between the two groups.

## Discussion

In this multicenter, international, retrospective, observational cohort study of patients with CVT, the rate of patients with cancer was 6.5%. We found that older age and an absence of referred headache to be independently associated with the presence cancer. Although involvement of superficial veins was numerically more frequent in patients with cancer, this association did not result being statistically significant (*p* = 0.067). Moreover, patients with CVT and cancer had a significantly higher risk of reaching the primary outcome defined as the composite of recurrent VTE and major hemorrhage, compared to patients without cancer. Similarly, the risks of major hemorrhage, ICH, and all-cause-death, and the composite of recurrent VTE and major hemorrhage and death were significantly greater in patients with cancer. Regarding recanalization rates, partial or complete, they were not significantly different between the two groups.

Data from the ISCVT and CErebral VEin Thrombosis International Study (CEVETIS) studies reported a 7.4% prevalences of malignancies [4, 20]. We also found a 6.5% prevalence of cancer in patients with CVTs.

Male gender, older age, and low BMIs were more frequent among patients with cancer. Males accounted for 53.7%, similar to a previous study reporting a rate of 55.6% [21]. Additionally, headache has been reported to be one of the most prevalent symptoms in patients with CVT, but it was significantly less frequent in subjects with cancer in our study. This finding supports the previous results of a sub-analysis performed on data from ISCVT study where Coutinho et al. [22] reporting a significantly higher rate of cancer in patients without headache compared to patients with headache (18% versus 6%, *p* = 0.01); especially for central nervous system (CNS) malignancy. Possible explanations for the presence of headache might include: more frequent isolated cortical vein thrombosis with lower risk for intracranial hypertension, the use of analgesics, as well as older age; in fact, the latter has been reported to be associated with a lower risk of headache in cerebrovascular diseases [23–26]. Indeed, we observed that cancer patients more frequently had thrombosis of the superficial venous system (85% in cancer versus 72% in no cancer). The sites of the thrombotic involvement in our cohort were similar to that reported in previous studies [4, 20, 27]. Our results may have implications for clinical practice. Indeed, the lack of headache in cancer patients with CVT could have led to a misdiagnosis or a delay in diagnosis and hence a delay in the initiation of treatment.

As further findings, patients with cancer were older, more frequently had chronic kidney disease, liver disease and were active smokers. As for additional VTE risk factors, we did not observe any significant differences between the two groups except for a less frequent family history of VTE and birth control use in cancer patients. This last sex-specific risk factor may be explained by the high prevalence of females and the young age in the cancer-free group. None of these factors resulted independently associated with cancer. Whether low BMI, liver disease, active smoking, involvement of a superficial vein, and absence of a family history of VTE could be useful indicators of increased risk for cancer diagnosis requires further study.

Concerning outcome results, we found that cancer patients had a more than three-fold higher risk of recurrent VTE or major bleeding compared to patients without cancer. Sensitivity analyses using propensity score matching and the 6-month analysis confirmed the results obtained in the main ones. The most remarkable finding was the high rate of major bleeding complications (22% patient-years in the cancer group and 6% patient-years in the non-cancer group), particularly of ICH (20% patient-years in the cancer group and 5% patient-year in the non-cancer group). However, it should be noted that rates of symptomatic ICH were similar (4.3 vs. 2.3% patient-years for cancer and non-cancer groups, respectively).

These findings differ from past literature where lower incidences of major bleeding, and of ICH, were observed in patients with VTE (range 3.8 to 4.0%) and in those with CVT and no-cancer (range 1.7 to 3.3%) [28, 29]. In our cohort, these differences can be explained by the cerebral localization of thrombosis in which venous infarction might have been associated with a high risk of bleeding. This risk can be increased by the presence of a local predisposing factors (e.g. brain tumors). However, due to a lack of data on the cancer sites, this hypothesis could not be tested. An important challenge to consider regarding intracranial hemorrhage in the context of CVT is that new or worsening hemorrhage may have been linked to progression of thrombosis and therefore may be more related to treatment efficacy rather than safety. Indeed, a further important goal in the management of patients with CVT is to promote recanalization, as it has been shown that lack of recanalization is also associated with long term morbidity [30]. In the present sub-analysis, recanalization was numerically less common in cancer patients compared to those without cancer, but the difference did not reach statistically significance. It could be hypothesized that this is due to the less use of VKA in patients with cancer compared to those without. However, the number of patients is too small to provide a specific comment on the different treatments. Moreover, the results from the main analysis of the ACTION-CVT study showed similar recanalization rates in patients treated with DOACs versus VKAs, and that treatment with DOACs was associated with a favorable safety profile when compared with VKA [13].

While the similar efficacy between DOACs and VKA is not surprising, the improved safety profile of DOACs over VKA is noteworthy [13]. This is in line with data showing the safety of DOACs over VKA in several non-CVT patient populations especially in the reduction of intracranial bleedings [31, 32].

We also found that cancer patients had a shorter follow-up and a substantially higher risk of death compared to patients without cancer. Nearly 60% had a follow-up of at least 90 days but data regarding the cause of death was not available.

Our study had several limitations including its retrospective design, along with non-central and non-blinded adjudication of events and imaging outcomes. However, baseline characteristics of the two groups were adjusted and matched through propensity score, but the possibility of residual bias cannot be excluded. Other limitations consist of the relatively small number of patients included in the cancer group which may have influenced the observed low event rate of recurrent VTE, in accordance with past studies [17, 29, 33]. This limited patient pool may have underpowered the analyses leading to overfitting in the multivariable model. Furthermore, we lacked information on the site, type, and stage of cancer which could have influenced the thrombogenic potential therein influencing outcome. Additionally, we lacked data on whether the cancer diagnosis was prior to or followed CVT. Information on cancer treatments, antineoplastic or surgical intervention, cancer remission, or withdrawal of care decisions during follow-up, which may have affected the risk of recurrence and decision to continue or stop anticoagulation were not available. Finally, it was not possible to perform a robust follow-up, so we do not know whether the increased death in the cancer group might have been related to CVT or the cancer itself. Finally, image acquisition may have been biased in patients with cancer, which may have contributed to higher asymptomatic hemorrhage rates. This however reflects real-world experience in patients with CVT and the cohort with cancer is the larger in the literature.

**Table 1:** Differences in baseline characteristics across patients with and without cancer

	Cancer (*n* = 67)	No cancer (*n* = 956)	*p*-value
Age (years), median (IQR)	59 (44–70)	43.5 (32–56)	< 0.001
Female gender, n/N (%)	31/67 (46.3%)	611/956 (63.9%)	0.004
Race, n/N (%)			
White	50/67 (74.6%)	659/949 (69.4%)	0.372
Black	11/67 (16.4%)	149/949 (15.7%)	0.876
Asian*	1/67 (1.5%)	39/949 (4.1%)	0.511
Other*	5/67 (7.5%)	102/949 (10.7%)	0.537
Ethnicity (% Hispanic)*, n/N (%)	4/64 (6.3%)	97/948 (10.2%)	0.392
Body mass index, mean ± SD	26.8 ± 7.0	29.5 ± 7.6	0.007
Chronic kidney disease*, n/N (%)	6/67 (9.0%)	33/955 (3.5%)	0.037
Liver disease*, n/N (%)	5/67 (7.5%)	16/955 (1.7%)	0.009
History of VTE, n/N (%)	12/67 (17.9%)	109/956 (11.4%)	0.111
Family history of VTE*, n/N (%)	1/67 (1.5%)	100/949 (10.5%)	0.010
Recent head trauma*, n/N (%)	7/67 (10.4%)	82/955 (8.6%)	0.652
Recent mastoiditis or sinusitis*, n/N (%)	7/67 (10.4%)	82/956 (8.6%)	0.651
Recent lumbar puncture*, n/N (%)	5/67 (7.5%)	41/956 (4.3%)	0.218
12 weeks post-partum*, n/N (%)	0/67 (0.0%)	38/947 (4.0%)	0.170
Birth control use*, n/N (%)	4/67 (6.0%)	231/938 (24.6%)	< 0.001
Active smoking, n/N (%)	16/67 (23.9%)	130/950 (13.7%)	0.021
Days from symptoms to diagnosis (median IQR)	2 (0–11)	4 (1–10)	0.100
Clinical presentation, n/N (%)			
Headache	31/67 (46.3%)	731/954 (76.6%)	< 0.001
Focal deficit	33/67 (49.3%)	367/955 (38.4%)	0.079
Seizure	16/67 (23.9%)	227/955 (23.8%)	0.984
Encephalopathy/Coma	19/67 (28.4%)	202/955 (21.2%)	0.166
Platelet count (x10^3 mm^3), mean ± SD	244.67 ± 117.11	266.23 ± 102.36	0.100
Creatinine (mg/dl), mean ± SD	0.89 ± 0.35	0.87 ± 0.46	0.657
One or more positive APL antibody*, n/N (%)	1/37 (2.7%)	81/801 (10.1%)	0.249
Factor V and/or prothrombin mutation*, n/N (%)	1/17 (5.9%)	46/661 (7.0%)	1.000
Imaging findings, n/N (%)			
Venous infarct	10/67 (14.9%)	263/952 (27.6%)	0.023
Cerebral edema	24/67 (35.8%)	293/952 (30.8%)	0.389
Intracranial hemorrhage	19/67 (28.4%)	369/952 (38.8%)	0.089
CVT location, n/N (%)			
Superficial vein only	57/67 (85.1%)	682/954 (71.5%)	0.016
Deep vein only*	5/67 (7.5%)	113/954 (11.8%)	0.329
Cortical vein only*	0/67 (0.0%)	30/954 (3.1%)	0.255
Superficial and deep*	5/67 (7.5%)	131/956 (13.7%)	0.191
Low molecular weight heparin, n/N (%)	36/67 (53.7%)	589/956 (61.6%)	0.201
DOAC, n/N (%)	31/67 (46.3%)	411/956 (43.0%)	0.601
VKAs, n/N (%)	22/67 (32.8%)	575/956 (60.1%)	< 0.001
Endovascular treatment*, n/N (%)	2/67 (3.0%)	87/956 (9.1%)	0.113
Neurosurgical treatment*, n/N (%)	6/67 (9.0%)	56/956 (5.9%)	0.288
Duration of treatment to imaging, (median IQR)	158.5 (75–256)	180 (96–310)	0.275

* Fisher’s Exact test performed

APL = anti-phospholipid; CVT = cerebral vein thrombosis; DOAC = direct oral anticoagulant; VKA = vitamin K antagonist

**Table 2:** Outcome events in patients with versus without cancer at 3 months

	Unadjusted	Model 1*	Model 2*	Propensity score matched
Primary outcome				
Recurrent VTE or major hemorrhage	*N* = 1023 OR 2.17 95% CI 1.18–3.99 *p* = 0.012	*N* = 835 aOR 3.87 95% CI 2.09–7.16 *p* < 0.001	*N* = 835 aOR 3.87 95% CI 2.06–7.25 *p* < 0.001	*N* = 835 aOR 2.93 95% CI 1.32–6.52 *p* = 0.008
Secondary outcome				
Recurrent VTE	*N* = 1023 OR 1.99 95% CI 0.87–4.56 *p* = 0.105	*N* = 836 aOR 1.11 95% CI 0.42–2.94 *p* = 0.831	*N* = 836 aOR 1.11 95% CI 0.42–2.90 *p* = 0.838	*N* = 836 aOR 1.62 95% CI 0.52–5.11 *p* = 0.407
Major hemorrhage	*N* = 1023 OR 2.29 95% CI 1.12–4.69 *p* = 0.023	*N* = 1019 aOR 3.70 95% CI 1.76–7.80 *p* = 0.001	*N* = 1019 aOR 3.75 95% CI 1.70–8.27 *p* = 0.001	*N* = 1018 aOR 3.56 95% CI 1.48–8.58 *p* = 0.005
ICH	*N* = 1023 OR 2.16 95% CI 1.03–4.56 *p* = 0.043	*N* = 1019 aOR 3.73 95% CI 1.73–8.03 *p* = 0.001	*N* = 1019 aOR 3.77 95% CI 1.69–8.41 *p* = 0.001	*N* = 1018 aOR 3.56 95% CI 1.42–8.91 *p* = 0.007
Symptomatic ICH	*N* = 1023 OR 1.25 95% CI 0.29–5.41 *p* = 0.767	*N* = 1019 aOR 0.87 95% CI 0.25–2.98 *p* = 0.825	*N* = 1019 aOR 0.88 95% CI 0.26–2.99 *p* = 0.834	*N* = 1018 aOR 1.33 95% CI 0.26–6.65 *p* = 0.732
All-cause-death	*N* = 1023 OR 14.90 95% CI 7.55–29.43 *p* < 0.001	*N* = 1019 aOR 7.56 95% CI 3.24–17.64 *p* < 0.001	*N* = 1019 aOR 8.10 95% CI 3.62–18.12 *p* < 0.001	*N* = 1018 aOR 11.33 95% CI 5.08–25.25 *p* < 0.001
Recurrent VTE or major hemorrhage or death	*N* = 1023 OR 4.45 95% CI 2.64–7.50 *p* < 0.001	*N* = 835 aOR 4.67 95% CI 2.58–8.44 *p* < 0.001	*N* = 835 aOR 4.74 95% CI 2.67–8.42 *p* < 0.001	*N* = 835 aOR 4.46 95% CI 2.18–9.14 *p* < 0.001
Partial/complete recanalization	*N* = 298 OR 0.53 95% CI 0.19–1.44 *p* = 0.213	*N* = 298 aOR 0.47 95% CI 0.14–1.60 *p* = 0.229	*N* = 298 aOR 0.48 95% CI 0.15–1.58 *p* = 0.230	*N* = 254 aOR 0.35 95% CI 0.07–1.60 *p* = 0.174

aOR = adjusted odds ratio; CI = confidence interval; ICH = intracranial hemorrhage; OR = odds ratio; VTE = venous thromboembolism

*Logistic regression analysis was adjusted for prespecified variables (Model 1) and for prespecified variables and variables that differed between the two groups as reported in Table S1 (Model 2). Propensity score matching was adjusted for prespecified variables and presence of headache

## Conclusion

Our results provide data supporting that older age and lack of headaches resulted being independently associated with cancer in patients with CVT. Additionally, patients with cancer had significantly worse outcomes compared to those without cancer. Our findings are in line with past studies, supporting the need for further investigation into the possible roles of these two factors.

### Electronic supplementary material

Below is the link to the electronic supplementary material.

Supplementary Material 1

Supplementary Material 2

## References

[1] Agnelli G (1997) Venous thromboembolism and cancer: a two-way clinical association. Thromb Haemost 78(1):117–120 PMID: 9198139 9198139 10.1055/s-0038-1657512

[2] Noble S, Pasi J (2010) Epidemiology and pathophysiology of cancer-associated thrombosis. Br J Cancer 102(Suppl 1):S2–9 20386546 10.1038/sj.bjc.6605599 PMC3315367

[3] Mahé I, Sterpu R, Bertoletti L, López-Jiménez L, Mellado Joan M, Trujillo-Santos J, Ballaz A, Hernández Blasco LM, Marchena PJ, Monreal M (2015) RIETE investigators. Long-term anticoagulant therapy of patients with venous thromboembolism. What are the practices? PLoS ONE 10(6):e0128741 26076483 10.1371/journal.pone.0128741 PMC4468159

[4] Ferro JM, Canhão P, Stam J, Bousser MG, Barinagarrementeria F, ISCVT Investigators (2004) Prognosis of cerebral vein and dural sinus thrombosis: results of the international study on cerebral vein and dural sinus thrombosis (ISCVT). Stroke 35(3):664–670 14976332 10.1161/01.STR.0000117571.76197.26

[5] Grisold W, Oberndorfer S, Struhal W (2009) Stroke and cancer: a review. Acta Neurol Scand 119(1):1–16 18616624 10.1111/j.1600-0404.2008.01059.x

[6] Martinelli I, De Stefano V (2010) Rare thromboses of cerebral, splanchnic and upper-extremity veins. A narrative review. Thromb Haemost 103(6):1136–1144 20352171 10.1160/TH09-12-0873

[7] Ferro JM, Canhão P, Aguiar de Sousa D (2016) Cerebral venous thrombosis. Presse Med 45(12 Pt 2):e429–e450 27816347 10.1016/j.lpm.2016.10.007

[8] Dentali F, Gianni M, Crowther MA, Ageno W (2006) Natural history of cerebral vein thrombosis: a systematic review. Blood 108(4):1129–1134 16609071 10.1182/blood-2005-12-4795

[9] Saposnik G, Barinagarrementeria F, Brown RD Jr, Bushnell CD, Cucchiara B, Cushman M, deVeber G, Ferro JM, Tsai FY, American Heart Association Stroke Council and the Council on Epidemiology and Prevention (2011) Diagnosis and management of cerebral venous thrombosis: a statement for healthcare professionals from the American Heart Association/American Stroke Association. Stroke 42(4):1158–1192 21293023 10.1161/STR.0b013e31820a8364

[10] Ortel TL, Neumann I, Ageno W, Beyth R, Clark NP, Cuker A, Hutten BA, Jaff MR, Manja V, Schulman S, Thurston C, Vedantham S, Verhamme P, Witt DM, Florez D, Izcovich I, Nieuwlaat A, Ross R, Schünemann SJ, Wiercioch H, Zhang W, Zhang Y (2020) American Society of Hematology 2020 guidelines for management of venous thromboembolism: treatment of deep vein thrombosis and pulmonary embolism. Blood Adv 4(19):4693–4738 33007077 10.1182/bloodadvances.2020001830 PMC7556153

[11] Prandoni P, Lensing AW, Piccioli A, Bernardi E, Simioni P, Girolami B, Marchiori A, Sabbion P, Prins MH, Noventa F, Girolami A (2002) Recurrent venous thromboembolism and bleeding complications during anticoagulant treatment in patients with cancer and venous thrombosis. Blood 100(10):3484–3488 12393647 10.1182/blood-2002-01-0108

[12] Chee CE, Ashrani AA, Marks RS, Petterson TM, Bailey KR, Melton LJ 3rd, Heit JA (2014) Predictors of venous thromboembolism recurrence and bleeding among active cancer patients: a population-based cohort study. Blood 123(25):3972–3978 24782507 10.1182/blood-2014-01-549733 PMC4064333

[13] Yaghi S, Shu L, Bakradze E, Salehi Omran S, Giles JA, Amar JY, Henninger N, Elnazeir M, Liberman AL, Moncrieffe K, Lu J, Sharma R, Cheng Y, Zubair AS, Simpkins AN, Li GT, Kung JC, Perez D, Heldner M, Scutelnic A, Seiffge D, Siepen B, Rothstein A, Khazaal O, Do D, Kasab SA, Rahman LA, Mistry EA, Kerrigan D, Lafever H, Nguyen TN, Klein P, Aparicio H, Frontera J, Kuohn L, Agarwal S, Stretz C, Kala N, El Jamal S, Chang A, Cutting S, Xiao H, de Havenon A, Muddasani V, Wu T, Wilson D, Nouh A, Asad SD, Qureshi A, Moore J, Khatri P, Aziz Y, Casteigne B, Khan M, Cheng Y, Mac Grory B, Weiss M, Ryan D, Vedovati MC, Paciaroni M, Siegler JE, Kamen S, Yu S, Leon Guerrero CR, Atallah E, De Marchis GM, Brehm A, Dittrich T, Psychogios M, Alvarado-Dyer R, Kass-Hout T, Prabhakaran S, Honda T, Liebeskind DS, Furie K (2022) Direct oral anticoagulants versus Warfarin in the treatment of cerebral venous thrombosis (ACTION-CVT): a multicenter international study. Stroke 53(3):728–738 35143325 10.1161/STROKEAHA.121.037541

[14] Liberman AL, Kamel H, Mullen MT, Messé SR (2016) International classification of diseases, Ninth Revision (ICD-9) diagnosis codes can identify cerebral venous thrombosis in hospitalized adults. Neurohospitalist 6(4):147–150 27695595 10.1177/1941874416648198 PMC5029556

[15] Handley JD, Emsley HC (2020) Validation of ICD-10 codes shows intracranial venous thrombosis incidence to be higher than previously reported. Health Inf Manag 49(1):58–61 30563370 10.1177/1833358318819105

[16] Salehi Omran S, Shu L, Chang A, Parikh NS, Zubair AS, Simpkins AN, Heldner MR, Hakim A, Kasab SA, Nguyen T, Klein P, Goldstein ED, Vedovati MC, Paciaroni M, Liebeskind DS, Yaghi S, Cutting S (2023) Timing and predictors of recanalization after anticoagulation in cerebral venous thrombosis. J Stroke 25(2):291–298 37282376 10.5853/jos.2023.00213 PMC10250867

[17] Lyman GH, Carrier M, Ay C, Di Nisio M, Hicks LK, Khorana AA, Leavitt AD, Lee AYY, Macbeth F, Morgan RL, Noble S, Sexton EA, Stenehjem D, Wiercioch W, Kahale LA, Alonso-Coello P (2021) American society of hematology 2021 guidelines for management of venous thromboembolism: prevention and treatment in patients with cancer. Blood Adv 5(4):927–974 33570602 10.1182/bloodadvances.2020003442 PMC7903232

[18] Bates SM, Greer IA, Hirsh J, Ginsberg JS (2004) Use of antithrombotic agents during pregnancy: the Seventh ACCP conference on antithrombotic and thrombolytic therapy. Chest. ;126(3 Suppl):627S-644S 10.1378/chest.126.3_suppl.627S 15383488

[19] Miranda B, Ferro JM, Canhão P, Stam J, Bousser MG, Barinagarrementeria F, Scoditti U (2010) ISCVT investigators. Venous thromboembolic events after cerebral vein thrombosis. Stroke 41(9):1901–1906 20634477 10.1161/STROKEAHA.110.581223

[20] Dentali F, Poli D, Scoditti U, Di Minno MN, De Stefano V, Siragusa S, Kostal M, Palareti G, Sartori MT, Grandone E, Vedovati MC, Ageno W; CErebral VEin Thrombosis International Study Investigators, Falanga A, Lerede T, Bianchi M, Testa S, Witt D, McCool K, Bucherini E, Grifoni E, Coalizzo D, Benedetti R, Marietta M, Sessa M, Guaschino C, di Minno G, Tufano A, Barbar S, Malato A, Pini M, Castellini P, Barco S, Barone M, Paciaroni M, Alberti A, Agnelli G, Giorgi Pierfranceschi M, Dulicek P, Silingardi M, Federica L, Ghirarduzzi A, Tiraferri E, di Lazzaro V, Rossi E, Ciminello A, Pasca S, Barillari G, Rezoagli E, Galli M, Squizzato A, Tosetto A (2012) Long-term outcomes of patients with cerebral vein thrombosis: a multicenter study. J Thromb Haemost. ;10(7):1297 – 302 10.1111/j.1538-7836.2012.04774.x 22578023

[21] Abelhad NI, Qiao W, Garg N, Rojas-Hernandez CM (2021) Thrombosis and bleeding outcomes in the treatment of cerebral venous thrombosis in cancer. Thromb J 19(1):37 34074321 10.1186/s12959-021-00292-9 PMC8171031

[22] Coutinho JM, Stam J, Canhão P, Barinagarrementeria F, Bousser MG, Ferro JM (2015) ISCVT investigators. Cerebral venous thrombosis in the absence of headache. Stroke 46(1):245–247 25378420 10.1161/STROKEAHA.114.007584

[23] Coutinho JM, Gerritsma JJ, Zuurbier SM, Stam J (2014) Isolated cortical vein thrombosis. Syst Rev case Rep case Ser Stroke 45:1836–1838 10.1161/STROKEAHA.113.004414 24743438

[24] Ferro JM, Canhão P, Bousser MG, Stam J, Barinagarrementeria F (2005) ISCVT investigators. Cerebral vein and dural sinus thrombosis in elderly patients. Stroke. 36:1927–1932 10.1161/01.STR.0000177894.05495.54 16100024

[25] Ferro JM, Melo TP, Oliveira V, Salgado AV, Crespo M, Canhão P et al (1995) A multivariate study of headache associated with ischemic stroke. Headache. 35:315–319 10.1111/j.1526-4610.1995.hed3506315.x 7635716

[26] Melo TP, Pinto AN, Ferro JM (1996) Headache intracerebral. Hematomas Neurol 47:494–500 10.1212/wnl.47.2.494 8757027

[27] Duman T, Uluduz D, Midi I, Bektas H, Kablan Y, Goksel BK, Milanlioglu A, Necioglu Orken D, Aluclu U, VENOST Study Group (2017) A Multicenter study of 1144 patients with cerebral venous thrombosis: the VENOST study. J Stroke Cerebrovasc Dis 26(8):1848–1857 28583818 10.1016/j.jstrokecerebrovasdis.2017.04.020

[28] Agnelli G, Becattini C, Meyer G, Muñoz A, Huisman MV, Connors JM, Cohen A, Bauersachs R, Brenner B, Torbicki A, Sueiro MR, Lambert C, Gussoni G, Campanini M, Fontanella A, Vescovo G, Verso M (2020) Caravaggio Investigators. Apixaban for the treatment of venous thromboembolism associated with cancer. N Engl J Med 382(17):1599–1607 32223112 10.1056/NEJMoa1915103

[29] Ferro JM, Coutinho JM, Dentali F, Kobayashi A, Alasheev A, Canhão P, Karpov D, Nagel S, Posthuma L, Roriz JM, Caria J, Frässdorf M, Huisman H, Reilly P, Diener HC, RE-SPECT CVT Study Group (2019) Safety and Efficacy of Dabigatran Etexilate vs dose-adjusted warfarin in patients with cerebral venous thrombosis: a randomized clinical trial. JAMA Neurol 76(12):1457–1465 31479105 10.1001/jamaneurol.2019.2764 PMC6724157

[30] Rothrock JF, Diener HC (2021) Headache secondary to cerebrovascular disease. Cephalalgia 41(4):479–492 33736481 10.1177/0333102421999045

[31] Carnicelli AP, Hong H, Connolly SJ, Eikelboom J, Giugliano RP, Morrow DA, Patel MR, Wallentin L, Alexander JH, Cecilia Bahit M, Benz AP, Bohula EA, Chao TF, Dyal L, Ezekowitz M, Fox AA, Gencer K, Halperin B, Hijazi JL, Hohnloser Z, Hua SH, Hylek K, Toda Kato E, Kuder E, Lopes J, Mahaffey RD, Oldgren KW, Piccini J, Ruff JP, Steffel CT, Wojdyla J, Granger D (2022) COMBINE AF (a collaboration between multiple institutions to Better Investigate Non-vitamin K antagonist oral anticoagulant use in Atrial Fibrillation) investigators. Direct oral anticoagulants Versus Warfarin in patients with Atrial Fibrillation: patient-level network meta-analyses of randomized clinical trials with interaction testing by age and sex. Circulation 145(4):242–255 34985309 10.1161/CIRCULATIONAHA.121.056355 PMC8800560

[32] van Es N, Coppens M, Schulman S, Middeldorp S, Büller HR (2014) Direct oral anticoagulants compared with vitamin K antagonists for acute venous thromboembolism: evidence from phase 3 trials. Blood 124(12):1968–1975 24963045 10.1182/blood-2014-04-571232

[33] Cheung YW, Middeldorp S, Prins MH, Pap AF, Lensing AW, Ten Cate-Hoek AJ, Villalta S, Milan M, Beyer-Westendorf J, Verhamme P, Bauersachs RM, Prandoni P, Einstein PTS, Investigators Group (2016) Post-thrombotic syndrome in patients treated with rivaroxaban or enoxaparin/vitamin K antagonists for acute deep-vein thrombosis. A post-hoc analysis. Thromb Haemost 116(4):733–738 27583311 10.1160/TH16-01-0041

