# Supplementary material for: Cerebral venous sinus thrombosis associated with cancer: analysis of the ACTION-CVT study
Source: J Thromb Thrombolysis. 2024 Jun 2;57(6):1008–17. doi: 10.1007/s11239-024-02997-w (PMC11315801; doi:10.1007/s11239-024-02997-w)
Supplement: Supplementary file 2 — Supplementary Material 2 [file 11239_2024_2997_MOESM2_ESM.docx]

**Supplementary material**

**Table S1.** Factors independently associated with cancer in patients with CVT.

**Table S2.** Primary outcome events in patients with versus without cancer according to competing-risk adjusted models.

**Table S3.** Outcome events in patients with versus without cancer at 6 months.

**Table S4.** Propensity score matching analysis at 6 months.

**Table S1. Factors independently associated with cancer in patients with CVT.**

|  | **Adjusted OR** | **95% CI** | **p-value** |
| --- | --- | --- | --- |
| *Age, per decade increase* | *1.28* | *1.08-1.52* | *0.005* |
| Sex | 0.75 | 0.43-1.31 | 0.312 |
| Body mass index | 0.96 | 0.92-1.01 | 0.081 |
| Chronic kidney disease | 1.58 | 0.59-4.20 | 0.364 |
| Liver disease | 2.87 | 0.93-8.83 | 0.066 |
| Family history of VTE | 0.23 | 0.03-1.72 | 0.152 |
| Birth control or hormone replacement therapy | 0.58 | 0.19-1.80 | 0.343 |
| Active smoking | 1.86 | 0.98-3.54 | 0.059 |
| *Headache* | *0.47* | *0.27-0.84* | *0.010* |
| Venous infarct* | 0.65 | 0.31-1.36 | 0.255 |
| Superficial Vein | 2.02 | 0.95-4.29 | 0.067 |

CI=confidence interval; CVT=cerebral venous thrombosis; OR=odds ratio; VTE=venous thromboembolism.

*detected at brain imaging tests.

**Table S2.** **Primary outcome events in patients with versus without cancer according to competing-risk adjusted models**

|  | **Competing risk** | **Competing risk Model 1** | **Competing risk Model 2** |
| --- | --- | --- | --- |
| *Primary outcome at 3 months* |  |  |  |
| Recurrent VTE or major hemorrhage | OR 2.93 95% CI 1.56-5.49 p=0.001 | aOR 4.42 95% CI 2.42-8.05 p<0.001 | aOR 4.38 95% CI 2.45-7.84 p<0.001 |
| *Primary outcome at 6 months* |  |  |  |
| Recurrent VTE or major hemorrhage | OR 2.88 95% CI 1.53-5.42 p=0.001 | aOR 4.23 95% CI 2.25-7.98 p<0.001 | aOR 4.22 95% CI 2.32-7.69 p<0.001 |

aOR=adjusted odds ratio; CI=confidence interval; OR=odds ratio; VTE=venous thromboembolism.

**Table S3. Outcome events in patients with versus without cancer at 6 months**

|  | **Unadjusted** | **Model 1** | **Model 2** |
| --- | --- | --- | --- |
| *Primary outcome* |  |  |  |
| Recurrent VTE or major hemorrhage | OR 2.03 95% CI 1.11-3.72 p=0.022 | aOR 3.57 95% CI 1.91-6.69 p<0.001 | aOR 3.57 95% CI 1.89-6.74 p<0.001 |
| *Secondary outcome* |  |  |  |
| Recurrent VTE | OR 1.81 95% CI 0.79-4.13 p=0.161 | aOR 1.01 95% CI 0.39-2.60 p=0.991 | aOR 1.00 95% CI 0.39-2.58 p=0.998 |
| Major hemorrhage | OR 2.19 95% CI 1.07-4.47, p=0.032 | aOR 3.52 95% CI 1.65-7.51, p=0.001 | aOR 3.57 95% CI 1.60-7.95, p=0.002 |
| ICH | OR 2.13 95% CI 1.01-4.48, p=0.047 | aOR 3.67 95% CI 1.71-7.90, p=0.001 | aOR 3.71 95% CI 1.66-8.29, p=0.001 |
| Symptomatic ICH | OR 1.15 95% CI 0.27-4.94 p=0.855 | aOR 0.80 95% CI 0.23-2.78 p=0.720 | aOR 0.80 95% CI 0.23-2.79 p=0.728 |
| All-cause-death | OR 16.33 95% CI 8.55-31.18, p<0.001 | aOR 8.62 95% CI 3.70-20.09, p<0.001 | aOR 9.48 95% CI 4.04-22.24, p<0.001 |
| Recurrent VTE or major bleeding or death | OR 5.02 95% CI 3.00-8.40 p<0.001 | aOR 5.15 95% CI 2.93-9.05 p<0.001 | aOR 5.28 95% CI 3.02-9.21 p<0.001 |
| Partial/complete recanalization | OR 0.52 95% CI 0.17-1.58 p=0.248 | aOR 0.34 95% CI 0.09-1.30 p=0.115 | aOR 0.38 95% CI 0.12-1.23 p=0.106 |

aOR=adjusted odds ratio; CI=confidence interval; ICH=intracranial hemorrhage; VTE=venous thromboembolism.

**Table S4. Propensity score matching analysis at 6 months.**

|  | **Propensity score matched** |
| --- | --- |
| Primary outcome |  |
| Recurrent VTE or major hemorrhage | aOR 2.68, 95% CI 1.21-5.94, p=0.015 |
| Secondary outcome |  |
| Recurrent VTE | aOR 1.44, 95% CI 0.46-4.55, p=0.530 |
| Major hemorrhage | aOR 3.26, 95% CI 1.37-7.77, p=0.008 |
| ICH | aOR 3.49, 95% CI 1.40-8.71, p=0.007 |
| Symptomatic ICH | aOR 1.14, 95% CI 0.24-5.54, p=0.868 |
| All-cause-death | aOR 13.58, 95% CI 6.23-29.58, p<0.001 |
| Recurrent VTE or major hemorrhage or death | aOR 5.35, 95% CI 2.59-11.04, p<0.001 |
| Partial/complete recanalization | aOR 0.44, 95% CI 0.12-1.68, p=0.229 |

aOR=adjusted odds ratio; CI=confidence interval; ICH=intracranial hemorrhage; VTE=venous thromboembolism.
